# Simulation of Static Ultrasonic Welding Based on Explicit Simulation and a More Accurate Representation of the Hammering Effect

**DOI:** 10.3390/ma19061213

**Published:** 2026-03-19

**Authors:** Filipp Köhler, Jan Yorrick Dietrich, Irene Fernandez Villegas, Clemens Dransfeld, David May, Axel Herrmann

**Affiliations:** 1 Composite Technology Center Stade/CTC GmbH (An Airbus Company), Airbus Straße 1, 21864 Stade, Germany; 2 Aerospace Structures and Materials Department, Faculty of Aerospace Engineering, Delft University of Technology, Kluyverweg 1, 2629 HS Delft, The Netherlands; 3 Faserinstitut Bremen e.V., Am Biologischen Garten 2, 28359 Bremen, Germany; dietrich@faserinstitut.de (J.Y.D.);; 4 Faculty of Production Engineering, University of Bremen, Badgasteiner Straße 1, 28359 Bremen, Germany

**Keywords:** thermoplastic composites, energy director, high-speed camera, neutral position

## Abstract

The utilisation of composite materials has the potential to play a vital role in the development of lightweight structures for future generations of aircraft, with the objective to reduce emissions. Ultrasonic welding is a process that has been proven to exhibit advantageous qualities, including the capacity to achieve welds with a comparatively short process time. Furthermore, its capacity to function as both a static and a continuous process makes it a viable candidate for facilitating the realisation of this objective. The present study investigates the potential of a novel explicit modelling approach for the static ultrasonic welding process to more accurately represent the welding process by incorporating a more precise representation of the hammering effect. The hammering effect describes the partial loss of contact between the sonotrode and the upper adherend. The model’s validation was achieved through a multifaceted approach that incorporates high-speed camera recording, encompassing digital image correlation, laser displacement sensor measurements, and static ultrasonic welding experiments. These experiments encompassed varying welding times, followed by fracture surface analysis. The findings showed that an explicit time-domain model can effectively represent the static welding process of unidirectional materials utilising a film energy director. The experimental validation demonstrated a high degree of correlation between the thermal behaviour of the welding interface and the simulation results. The study demonstrated that the neutral position of the sonotrode exhibited an increase during the initial phase of the welding process due to dynamic stresses. This phenomenon enables reduced constraint movement of the adherends and the energy director, which results in the disconnection of the sonotrode from both the upper adherend and the energy director, as well as the adherends and the anvil. The higher neutral position of the sonotrode was then implemented in an explicit simulation of the static ultrasonic welding process.

## 1. Introduction

The aerospace industry invariably places significant emphasis on the concept of lightweight design. This focus is set to assume even greater significance for the forthcoming generations of aircraft, as the aerospace industry endeavours to achieve net-zero emissions and develop innovative propulsion systems that utilise, for instance, liquid hydrogen. It is imperative to minimise liquid hydrogen consumption during operation in order to reduce the weight of the storage system, given the greater complexity of storing liquid hydrogen in comparison with traditional fuels.

The utilisation of composite materials, which possess distinctive properties, holds considerable potential in this regard. Special attention should be given to thermoplastic composites as they combine the lightweight potential with the possibility of new designs due to innovative manufacturing and rivetless assembly technologies. Ultrasonic welding can be one of those technologies. The technology allows short welding times and low energy consumption. Ultrasonic welding is based on low-amplitude and high-frequency vibrations applied transversally to the welding surface through a sonotrode [1,2]. Frictional and viscoelastic heating are responsible for heat generation mainly in the welding interface where a so-called energy director is placed [3]. The energy director undergoes higher cyclic strains due to its lower stiffness compared to the adherends and, therefore, heat generation is concentrated there [4,5]. The energy director is usually a neat polymer element and can have different forms. There are triangular protrusions moulded on of the adherends [6], thin polymer films [5] or woven meshes [7]. Ultrasonic welding can be applied as a static or continuous process. The main difference lies in the relative movement between the adherends and the sonotrode, which is characteristic of the continuous process.

Different research was conducted on the static and continuous ultrasonic welding process. However, research on the simulation of ultrasonic welding is mostly limited to the static process.

Levy et al. [3,8,9] developed a simulation model which incorporates frictional and viscoelastic heating and the hammering effect. The hammering effect describes the partial loss of contact between the sonotrode and the upper adherend. Nonetheless, their simulation is limited to heat generation in the interface and calculations performed in the frequency domain using an analytically derived term. For heat generation in the interface, they assumed full amplitude transmission into the upper adherend which results in the corresponding cyclic strain in the welding interface which is derived from a FEM simulation. The hammering effect is incorporated as a coefficient representing the time the sonotrode is not in contact with the upper adherend. The coefficient is used to fit the total dissipated energy of the model to the experimental result.

In addition, Yang et al. [10] proposed a modelling approach based on the harmonic balance method which they adopted from ultrasonic metal welding with a vibration direction of the sonotrode parallel to the welding surface. Within the simulation, repeated separation and collision of the workpieces (times where the adherends were not in contact) was considered for the early stage of the process, and were considered as the cause of friction heating. This collision–separation cycle faded out when the adherends started to stick to each other. Consequently, frictional heating also decreased and was superposed by viscoelastic heating. Similar to Levy et al. [3,8,9], they considered full amplitude transmission into the upper adherend and simulation in the frequency domain.

Tutunjian et al. [11,12] showed a different simulation approach using explicit FEM analysis. They were able to show that frictional heating at the edges is significantly larger than in the centre. However, their welding approach uses an anvil which is smaller than the sonotrode and does not require any energy director. This is not the case in most other applications of ultrasonic welding.

Further explicit simulation approaches were developed by Li et al. [13] and Tan et al. [14]. While Li et al. combined the simulation with an artificial neural network to predict failure loads, Tan et al. used a similar modelling approach to Tutunjian et al. They performed simulation and validation experiments for spot welding of CF/PA66 without the use of an energy director. Instead of using the nominal amplitude set in the welding machine, actual vibrations were measured using a high-frequency displacement sensor. The real-time data was applied to the tip of the sonotrode within the FEM simulation. To consider recurring loss of contact between the sonotrode and the upper adherend, a constant hammering coefficient, following the approach developed by Levy et al. [3,8,9], was introduced. The simulation showed good correlation with the experimental results derived within this study.

Rao et al. [15] presented an alternative simulation model of CF/PA6 and pure PA6. They developed a 3D thermomechanical model in Comsol. They concluded that the presence of fibres in the adherends lead to more heat flux from the welding interface into the adherends due to the higher thermal conductivity of the fibres in fibre direction compared to the polymer. It must be noted that this is only valid for short fibre composites where fibres are freely oriented in all dimensions. Continuous fibre-reinforced composites usually have no fibres oriented in thickness direction, thus resulting in low thermal conductivity in that direction.

Zweifel et al. [16]. focussed on the simulation of ultrasonic-assisted insertion of pins in sandwich structures. Although the process uses different types of joining partners, the underlying heating mechanisms are comparable to the joining of two monolithic fibre-reinforced composites. Their explicit simulation model was validated by experiments and showed good correlation with a high prediction rate of the final connection quality. Although they incorporated the hammering coefficient as a constant value, similar to Levy et al. [3,8,9] they determined it based on data from the actual simulation and the validation experiments. They tracked the position of the sonotrode and the pin in the FEM model during experiments and then defined time durations of contact and non-contact. The ratio between the two resulted in the hammering coefficient which is still limited to loss of contact between the sonotrode and the pin only.

A phenomenon which has currently been studied only through experimental research is through-the-thickness heating (TTH), especially of the upper adherend. TTH leads to excessive heating of the upper adherend and defects in the welded parts such as deconsolidation, affecting the mechanical performance. Jongbloed et al. [17] already showed that this cannot be attributed to heat conduction from the welding interface only. Viscoelastic bulk heating was suspected to be one of the additional causes. This assumption was based on experimental observations and a heat transfer model where the interface temperatures were given as inputs based on the experimental results. This model did not, however, represent the actual heat generation by frictional and viscoelastic heating and did not include the hammering effect which can play a role in the heat generation within the adherends.

The mentioned studies on the different simulation approaches are performed either in the frequency or the time domain. They all incorporate frictional and viscoelastic heating. They also partially consider the hammering effects. In most cases this is achieved by applying a constant coefficient. Despite the fact that in some simulation models the hammering effect is already incorporated based on actual measurement data instead of a constant coefficient, the consideration is limited to the interaction of the sonotrode and the upper adherend.

In our previous publication [18], we showed that the hammering effect is not limited to the loss of contact between the sonotrode and the upper adherend but also leads to loss of contact between the adherends, the energy director and even between the lower adherend and the anvil. Furthermore, we showed that the amplitude is not fully transmitted into the adherends and that the sonotrode and adherends are not in harmonic resonance.

This behaviour is not reflected in the current aforementioned simulation approaches. Therefore, this paper investigates how a new explicit modelling approach for the static ultrasonic welding process can more accurately represent the welding process by including a more accurate representation of the hammering effect through physical representation of the actual movement of the sonotrode, adherends and energy director and their interactions. The model is validated by high-speed camera recordings including image correlation, laser displacement sensor measurements and static ultrasonic welding experiments using different welding times and subsequent fracture surface analysis. Fracture surfaces were analysed to assess the affected area and compare it to the results of the simulation.

## 2. Material and Methods

### 2.1. Material

The material used in this study was Toray Cetex^®^ TC1225 unidirectional prepreg tape from Toray Advanced Composites (TAC, Nijverdal, The Netherlands). The material was delivered as already press-consolidated laminates by the supplier. The laminates were composed of 12 plies with the stacking sequence (45/0/135/0/90/0)s. They were inspected by ultrasonic C-scan for voids and delaminations. Ultrasonic welding specimens (100 mm × 25 mm) were extracted from areas without defects using water jet cutting. Samples were degreased and cleaned with isopropyl alcohol prior to welding.

The used energy director was made of VICTREX LMPAEK^TM^ (Victrex, Lancashire, UK), the same polymer as used in the prepreg material, and was delivered as amorphous films with a thickness of 0.2 mm. The energy directors were cut to 25 × 35 mm^2^ pieces to be larger than the intended welding area. For the DMA analysis, 2 mm thick plates made of VICTREX LMPAEK™ polymer were supplied and tempered to fully crystallise according to manufacturer specifications at 200 °C.

### 2.2. Static Ultrasonic Welding and Evaluation

A 20 kHz micro-processor controlled ultrasonic welder from Herrmann Ultraschalltechnik, Germany, was used to weld specimens into a single lap shear configuration with an overlap of 12.5 mm. In the static ultrasonic welding setup used in this study, a welding head provided with a rectangular sonotrode with dimensions of 14.9 × 27 mm^2^ was used. The welding head was mounted onto a Kuka KR 500 robot (Augsburg, Germany) equipped with a Kuka KR C5 controller.

The ultrasonic welder had a maximum power output of 4.2 kW and automatically adjusted the power to keep the vibration amplitude constant. The crest-to-trough amplitude was set to 85 µm.

The welding force Fw was set to 500 N, corresponding to a welding pressure of 1.6 MPa for the contact area of the sonotrode (12.5 × 25 mm^2^). A consolidation time of 200 ms using 500 N was used. This did not result in properly consolidated welds. The intended measure to compare the experimental and simulation results was the affected area of the energy director, e.g., the amount of energy director that shows visible crystallisation or melting. Therefore, heat dissipation during the consolidation phase had to be avoided.

**Figure 1:**
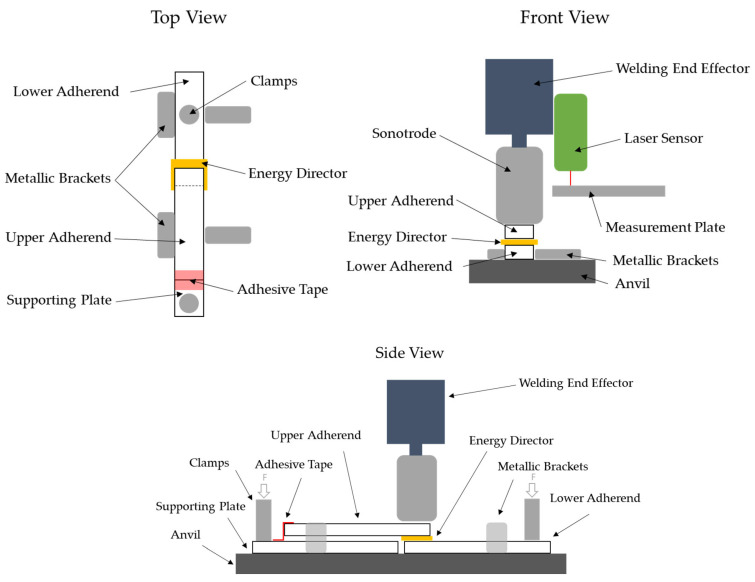
Custom-made welding jig which prevents the specimens from moving during the welding operation, including laser displacement sensor for measurement of vertical displacement.

Time-controlled welding was performed. 10 different welding times were used throughout the study: 100 ms, 125 ms, 150 ms, 175 ms, 200 ms, 250 ms, 300 ms, 350 ms, 400 ms and 500 ms. Six repetitions per set of welding parameters were performed. The samples were welded in a single overlap configuration (Figure 1) with an overlap of 12.5 mm, resulting in an intended welding area of 12.5 × 25 mm^2^.

**Figure 2:**
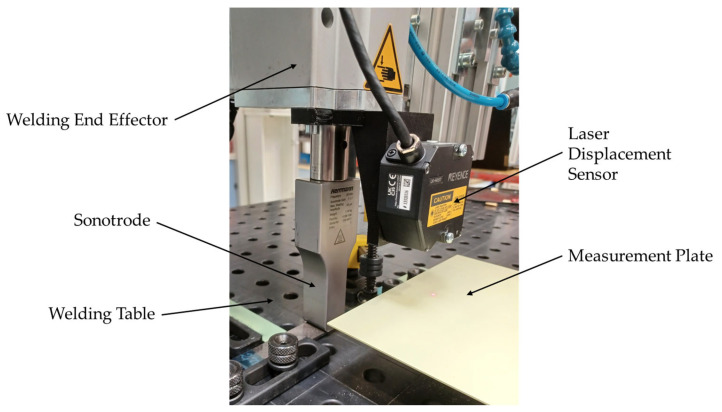
Welding setup used throughout the study. The welding head included the ultrasonic welder and the laser displacement sensor mounted to a Kuka robot. The measurement plate is fixed to the anvil.

The welding head was equipped with a Keyence laser displacement sensor LK-H057 (Frankfurt am Main, Germany) with a repeatability of 0.025 µm to measure the vertical displacement of the sonotrode during the welding process (Figure 2). Measurements were performed against a measurement plate which was not affected by the welding process. The laser displacement sensor moved together with the sonotrode and, at the beginning of the vibration phase of the welding process, displacement was set to zero to only measure relative movement during the welding process.

The specimens were held in place by a custom-made jig as shown in Figure 1. The jig prevented the specimens from moving in plane during the welding process and allowed for free access to the weld area. The jig represents the same clamping situation as used in the simulation.

**Figure 3:**
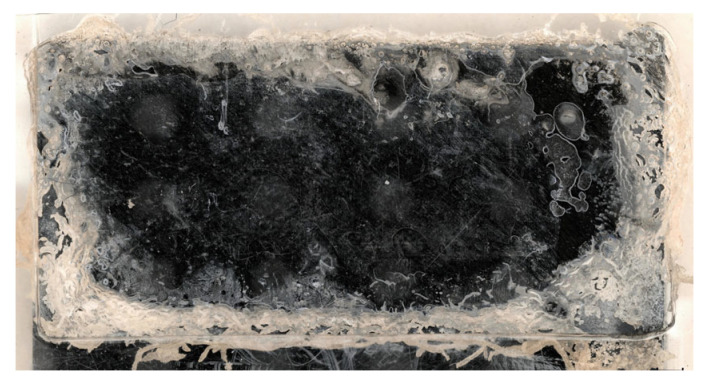

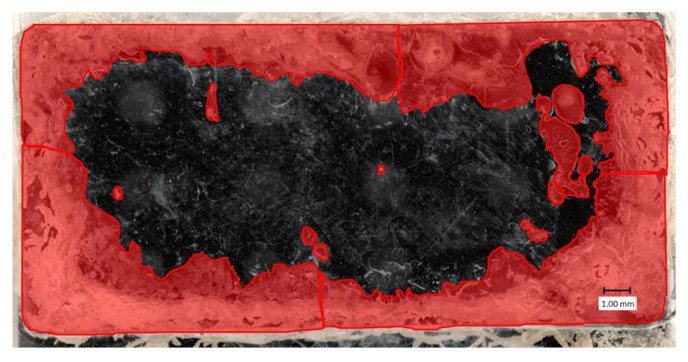
Example measurement of the affected area based on the fracture surface using a stereomicroscope for 200 ms welding time. The red area marks the part of the total overlap where the energy director reached Tg, crystallised or was molten (affected area). Left image shows the original fracture surface before measurement.

The welded samples were tested under tensile loading following the ASTM D1002 [19] in a Zwick/Roell Z250 universal testing machine (Ulm, Germany). The hydraulic grips of the testing machine were offset to ensure parallelism between the weld line and the load path. The fracture surfaces were visually inspected using a Keyence VHX-X1F digital microscope to assess the amount and pattern of molten or crystallised energy director. In the fracture surfaces, a pattern of half-transparent white dots are visible which can be attributed to an artefact caused by the microscope itself, and can therefore be disregarded in the interpretation of the results. Visual inspection only allows the assessment of changes in the appearance of the energy director (from transparent (amorphous) to opaque (crystallised)), which cannot be differentiated if the energy director reaches melting temperature or only cold crystallisation temperature, which is about 210 °C. Here, for simplification, we generally refer to both changes as “affected area”. Figure 3 shows a representative measurement of the affected area on the fracture surface of the lap shear coupons. The affected area is given as a percentage of the actual overlap for each coupon, to compensate for cutting and positioning tolerances. The results were compared to the simulation results using the same welding times.

### 2.3. Material Characterisation

**Figure 4:**
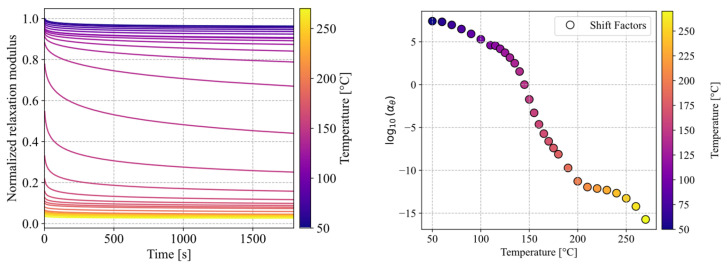
Normalised relaxation modulus of a representative sample of VICTREX LMPAEK™, ranging from 50 °C to 270 °C, and respective shift factors.

The viscoelastic characterisation of the VICTREX LMPAEK™ polymer was conducted with a TA Q800 DMA (TA Instruments, New Castle, DE, USA). Specimens were cut into 30 × 10 × 2 mm^3^ and tested with a single cantilever beam setup at 1% strain. The results are shown in Figure 4.

The frictional coefficient between the flat ED and the adherends was tested according to DIN EN ISO 8295 at room temperature and was found to be µ=0.25. The energy director film ($63\;\times \;63\;{mm}^{2}$) was adhesively bonded to a steel specimen slider. The specimen slider was pulled over the composite part at $F_{N}=\;21\;N$ using a winch pulley at a testing speed of v=100 mm/min. The force F_T_ was measured using a Zwick/Roell Z250 universal testing machine.

### 2.4. High-Speed Camera Experiments and Digital Image Correlation

**Figure 5:**
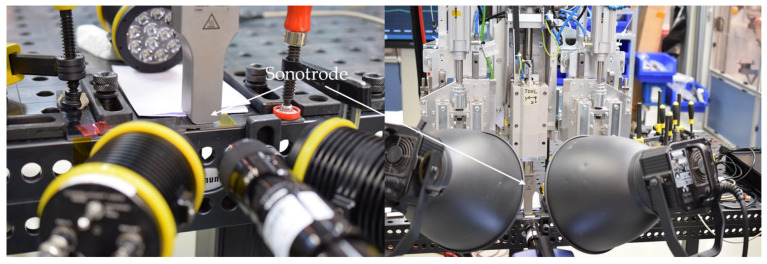
Continuous light recordings with Photron camera at 1 × 10^5^ FPS (**left**), and flash videography with Shimadzu camera at 1 × 10^6^ FPS (**right**) [18].

**Figure 6:**
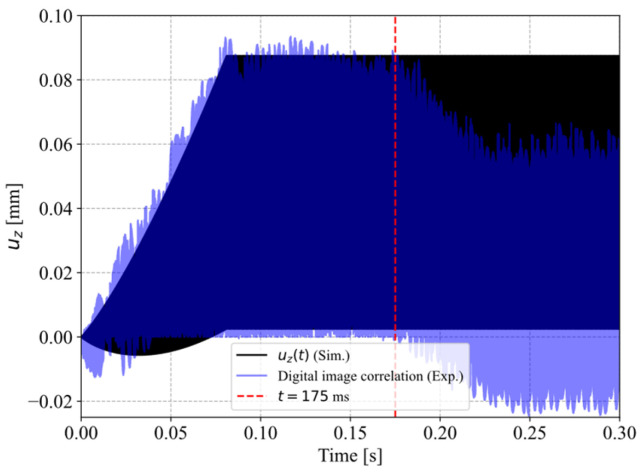
Vertical translation of the sonotrode as measured at 100,000 FPS and estimated via digital image correlation (blue), and as implemented in Abaqus (black).

High-speed recordings of the process were made by the Fraunhofer Institute of High-Speed dynamics, Ernst Mach Institute (EMI), from Efringen Kirchen, Germany. A Photron FASTCAM NOVA S-16 (Reutlingen, Germany) was used to record both at 1 × 10^5^ and 1.5 × 10^5^ frames per second (FPS) according to Figure 5 left. The recordings resulted in 384 × 240 px at 8.2 ms and 256 × 240 px at 4.9 µs shutter speed, using a Navitar Distanz Makro Zoom Optik 12X (Rochester, NY, USA). Secondly, a Shimadzu HPV-X (Duisburg, Germany) was used with the same optics to record at 1 × 10^6^ FPS, resulting in a resolution of 400 × 250 px at a shutter speed of 500 ns (Figure 5 right). For the Photron recordings, permanent LED light sources were used, while for the Shimadzu recordings, a timed flash-light was adapted, as short exposure times led to insufficient brightness. Due to the Shimadzu technology framework, recordings at 1 × 10^6^ FPS were limited to 128 frames in total, resembling a time span of 0.128 ms. The recording was made 81 ms after initiation of the welding process, when full amplitude build-up was achieved (compare Figure 6).

The recordings of different welds were analysed via digital image correlation (DIC) using Tracker software (Version 6.2.0) from physlets.org, with an evolution rate and tether of 0% and an acceptance level of 1. By minimisation of the squared error of the RGB values between a defined mask and a search mask, this process allows for efficient tracking of moving parts, even allowing for sub-pixel interpolation.

Figure 6 compares the vertical translation of the sonotrode during welding trials measured at 1 × 10^5^ FPS using the high-speed camera in blue and the implemented translation of the sonotrode in the simulation model in black. The graph shows a gradual build-up of the amplitude until 0.081 s, reaching a plateau of vibration around a sonotrode neutral position of 0.045 mm. After a duration of 0.175 s a gradual decrease in the neutral position occurs. We define the neutral position of the sonotrode as the point of the sonotrode sine wave vibration where the amplitude is zero.

### 2.5. Laser Displacement Sensor Data

**Figure 7:**
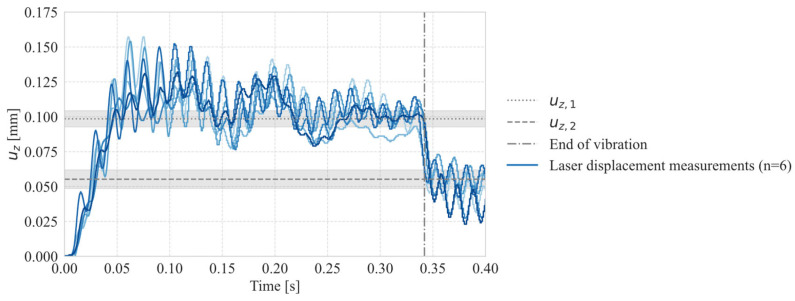
Change in neutral position: Uz, 1 was defined via the average of the plateau in uz (30 ms before the end of the vibration phase), averaged over all laser displacement measurements at that welding time. Uz, 2 was defined as the average (30 ms after the end of the vibration phase) of the second plateau (**left**).

Figure 7 shows the laser displacement sensor measurement data for welds using a welding time of 350 ms, which is equal to the simulation time of the model. The data was used to quantify the already observed neutral position of the sonotrode. The graph shows the vertical displacement of the sonotrode compared to the measurement plate. For the determination of the neutral position of the sonotrode implemented in the simulation model the data for all experiments at this welding time were averaged. The increase in neutral position was defined as the difference ($\Delta \;u_{z}$) between the displacement 30 ms before the end of the vibration phase u_z,1_ and 30 ms after the end of the vibration phase u_z,2_. The values were chosen to account for the periodic oscillation of the measurement data. The average value is therefore 0.043 µm with a standard deviation σ of 0.009 µm.

## 3. Modelling

The process simulation was created within the framework of an explicit fully coupled temperature–displacement analysis in Simulia Abaqus. Abaqus explicit is based on the dynamic equilibrium, which is derived from Newton’s second law of motion and numerically implemented as:

(1) $$\underline{M}\;\ddot{u}(t)=P(t)-I(t),$$

with $\underline{M}$ being the nodal lumped mass matrix, P(t) being the external forces vector and I(t) being the internal forces vector. As this approach underlies the Courant–Friedrichs–Lewy (CFL) criterion, the minimum stable time increment for forwards integration through time is dependent on the smallest element size and the speed of sound in the material, which itself is a function of the time- and temperature-dependent elastic properties. This stability criterion leads to large computation demands but also allows for an accurate representation of the conservation of momentum. This way, the high-speed interaction of the sonotrode and the adherends can be represented and the hammering effect, i.e., their loss of contact, can be implemented inherently through the conservation of momentum with no need to explicitly define hammering via the hammering coefficient.

### 3.1. Material Modelling

In order to implement the self-heating phenomena, two heating mechanisms were embodied. The frictional heating was implemented via surface-to-surface interactions and the *GAP HEAT GENERATION module in combination with the Coulomb *PENALTY friction contact sub option, using a frictional coefficient of μ=0.25 at room temperature in accordance with Levy et al. [3], and linearly decreasing to μ=0 at melting temperature, assuming no frictional heating was present when the energy director was molten. As Abaqus does not allow the friction coefficient to be defined as a function of the individual surface temperatures involved in contact, a simplified approach was adopted: the temperature at which the friction coefficient linearly decreases to μ = 0 was therefore set to the average interface temperature, representing the condition where the energy director reaches its melting temperature while the adherends remain cooler. The second heating mechanism, i.e., viscoelastic heating, was implemented via the *INELASTIC HEAT FRACTION material option using a heat fraction coefficient of 1, which allowed conversion of all of the inelastic mechanical energy from the *VISCOELASTIC material definition into a volumetric heat source in the fully coupled temperature–displacement framework.

In order to cover the viscoelastic material behaviour in a time-domain explicit simulation, the relaxation behaviour of the material was captured in a DMA Q800 using 1% strain in a single cantilever beam setup at temperatures ranging from 50 °C to 275 °C in steps of 5–10 K. This way, a smooth master curve at 145 °C was assembled representing the material behaviour over a long range of time, from 2 × 10^−7^ s to 9 × 10^18^ s, which can be shifted to the desired temperature within the framework of time-temperature super positioning (TTS). The master curve was fitted with a n = 12 term Prony series ($R^{2}=0.9996)$ using a Python (Version 3.12) script (see Figure 4) and implemented via the *VISCOELASTIC material option (Equations (3)–(5)); it was then combined with the *TRS (temperature-time-shift) suboption for the Williams–Landel–Ferry (WLF) approximation of shift factors, which allows interpolatation between the respective horizontal shift factors used for TTS-shifting of the master curve. In the case of deviatoric stress tensor components ${\tilde{\tau }}_{i}$, they are calculated via

(2) $${\tilde{\tau }}_{i}=\frac{{\hat{g}}_{i}^{P}}{\tau_{i}^{G}}\int_{0}^{t}e^{-\xi^{\prime }\left(\tau \right)\tau_{i}^{G}}{\tilde{\tau }}_{0}\left(t-\tau \right)d\tau ,$$

while hydrostatic counterparts $\phi_{i}$ are calculated via

(3) $$\phi_{i}=\frac{{\hat{k}}_{i}^{P}}{\tau_{i}^{K}}\int_{0}^{t}e^{-\xi^{\prime }\left(\tau \right)\tau_{i}^{K}}\phi_{0}\left(t-\tau \right)d\tau ,$$

with ${\hat{g}}_{i}^{P}$, $\tau_{i}^{G}$ and ${\hat{k}}_{i}^{P}$, $\tau_{i}^{K}$ being the respective Prony parameters, ${\tilde{\tau }}_{0}$ and $\phi_{0}$ being the respective instantaneous stress tensors components, τ being the relaxation time and $\xi^{\prime }$ being the shifted relaxation time following

(4) $$\xi^{\prime }\left(\tau \right)=\int_{0}^{\tau }\frac{dt^{\prime }}{\alpha_{T}\left(T\left(t^{\prime }\right)\right)},$$

where $\alpha_{T}$ are the shift factors for each temperature T, described by an WLF-approximation:

(5) $$log\;\alpha_{T}=\frac{-C_{1}(T-T_{ref})}{C_{2}+(T-T_{ref})}.$$

**Figure 8:**
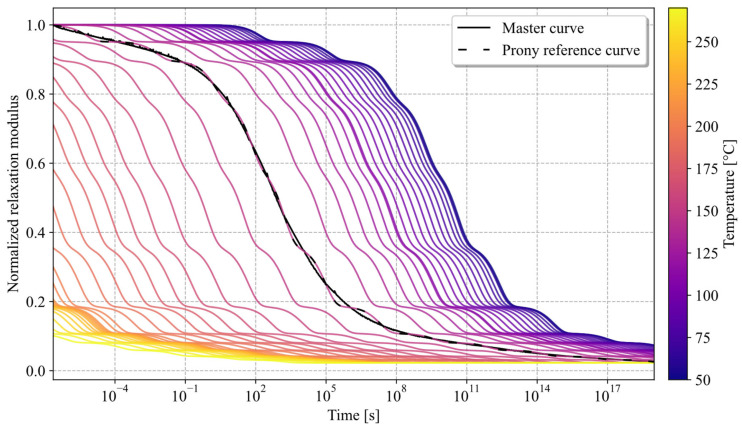
Normalised shifted relaxation moduli of VICTREX LMPAEK™ in Prony representation using shift factors obtained during master curve assembly.

These shift factors were derived directly from the assembly of the time-domain master curve (Figure 8) using the individual relaxation experiments in TA Trios software (Version 5.11.0.31), and later used to derive the WLF parameters of $C_{1}=21.164,\;C_{2}=54,9813\;K\;at\;T_{ref}=145\;{}^{\circ}C$ ($R^{2}=\;0.992$, evaluated between 145 °C and 275 °C). As most literature is based on a frequency domain modelling, it is noteworthy that this TTS-shift only implicitly reflects high frequency loading—as the shifting is performed in the time domain, experiments at different temperatures were used to extrapolate relaxation in the time domain rather than explicitly shifting loss and storage modulus.

The self-heating from a time-domain relaxation approach is mathematically equivalent to the respective frequency domain heating when assuming a harmonic sinusoidal loading, which was also confirmed by a one-element model. The relaxation behaviour, itself described via the Prony parameters ${{\hat{g}}_{i}}^{P}$ and $\tau_{i}$, can be transformed via Fourier transformation into the loss modulus at a given frequency as

(6) $$G^{\prime \prime }\left(\omega \right)=\frac{{{\hat{g}}_{i}}^{P}\tau_{i}\;\omega }{1+{\tau_{i}}^{2}\omega^{2}}.$$

The *VISCOELASTIC material option for the energy director material was used in combination with *ELASTIC to define the instantaneous modulus of $E_{0}=2.8$ GPa and ν = 0.39. In order to save computational efforts, the composite adherends were homogenised to a macro level, making use of classical laminate theory, and defined as engineering constants via *ELASTIC.

**Figure 9:**
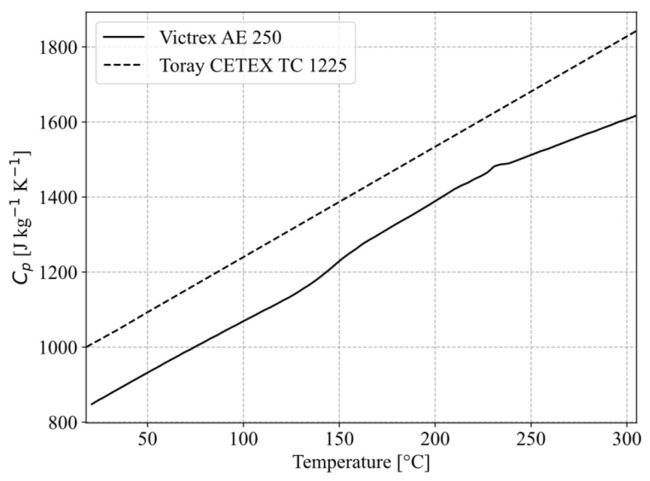
Specific heat capacity of VICTREX LMPAEK™, extrapolated from [20], and Toray Cetex^®^ TC1225, provided by manufacturer.

Thermally, the simulation embodies temperature-dependent material properties for specific heat capacity (Figure 9) and thermal conductivity (Table 1) for both energy director and adherend materials. The fibre-reinforced adherends are described by equivalent orthotropic values, which are calculated from individual layer orthotropic values using transformation and homogenisation:

(7) $$\lambda_{xx}\left(\theta \right)=\lambda_{11}\;cos^{2}\theta +\lambda_{22}\;sin^{2}\theta ,$$

(8) $$\lambda_{yy}(\theta )=\lambda_{11}\;{sin}^{2}\;\theta +\lambda_{22}\;{cos}^{2}\;\theta ,$$

(9) $$\lambda_{eq.}=\frac{1}{N}\sum_{i=1}^{N}\lambda .$$

Temperature-dependent specific heat capacities were used for VICTREX LMPAEK™, which was extrapolated from [20], and Toray Cetex^®^ TC 1225, respectively—for both materials, melting enthalpies were neglected.

To ensure realistic thermal behaviour, thermal contact is allowed between all parts via surface-to-surface interactions (heat transfer coefficient $h_{c}=2000\;Wm^{-2}K^{-1}$) [21] and a surface film condition ($h_{c}=5\;Wm^{-2}K^{-1}$) is applied to all free surfaces, reflecting free convection.

The materials’ densities are kept constant at $\rho =1.58\;g\;cm^{-3}$ for Toray Cetex^®^ TC 1225 and $\rho =1.3\;g\;{cm}^{-3}$. For all metallic parts, constant material properties of stainless steel were assumed: $\rho =7.8\;g\;{cm}^{-3},\;E=210\;GPa,\;k=16.2\;Wm^{-1}K^{-1}\;and\;c_{p}=500\;J\;{kg}^{-1}\;K^{-1}$.

### 3.2. Process Modelling

**Figure 10:**
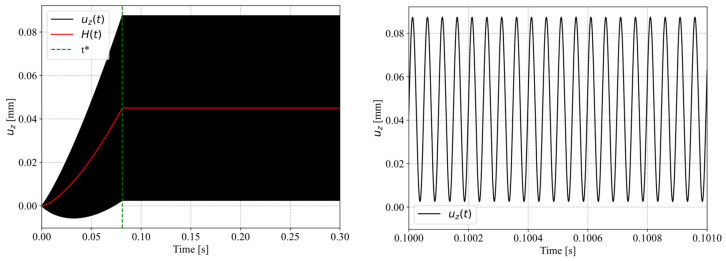
Amplitude definition of the vertical sonotrode tip displacement from 0 to 300 ms (**left**), and zoomed-in at 100 ms (**right**). On the left the neutral position is marked as a red line.

The main boundary condition, which is the movement of the sonotrode, was derived via the digital image correlation of the high-speed footage, following

(10) $$u_{z}\left(t\right)=\{\begin{array}{c} (m_{\alpha }\;t)\;sin(\omega t)+H(t)\;at\;t\leq t^{*}\\ \alpha_{sono}\;sin(\omega t)+H(t)\;at\;t>t^{*} \end{array},$$

and for H, which is the neutral position, and h the constant part of the neutral position, according to Figure 6:

(11) $$H\left(t\right)=\{\begin{array}{c} {(h/\left({t^{*}}^{n})\right)}^{n}\;at\;t\leq t^{*}\\ h\;at\;t>t^{*} \end{array},$$

which was consequently implemented via a *VUAMP user routine, enforcing a Dirichlet-type displacement boundary condition acting on the sonotrode tip. It reflects a harmonic oscillation with a circular frequency of ω=125,663.7 Hz (20 kHz), growing to a target amplitude of $\alpha_{sono}=0.0425\;mm$ linearly (slope $m_{\alpha }=\alpha_{sono}/t^{*}\approx 0.52\;mms^{-1}$) until 81 ms of process time, the time where target amplitude is reached, and then remains constant. Additionally, the neutral position of the oscillation grows exponentially (n = 1.5), ensuring a smooth transition, ultimately reaching $H(t>t^{*})=0.045\;mm$, as derived from laser-displacement measurements (Figure 7). This does not reflect the gradual decrease in the neutral position as shown in Figure 6 because the model does not include any plasticity. Figure 10 shows the boundary condition of the sonotrode amplitude model.

**Figure 11:**
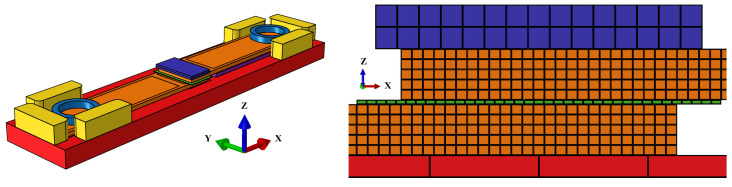
Geometry and mesh used for simulation (**left**) and close-up of welding stack (**right**), with nodes used for vertical displacement analysis highlighted in red. The adherends are orange, the energy director is green, the sonotrode is dark blue, the anvil is red, the stops are yellow, the crew clamps are light blue, and the support plate is purple.

Lastly, no mass scaling was used, as early studies showed a high influence of artificial scaling on both the self-heating phenomenon and the mechanical interactions. A mesh study was adopted, resulting in a mesh with local refinement which showed convergence of the solution of three full amplitude oscillations (<5% change between differently meshed models) with respect to the global energy outputs ALLCD and ALLFD, resembling the overall viscoelastic and frictional dissipation, respectively. The model comprises a total of 129,072 linear hexahedral elements of type C3D8T. The energy director is composed of 2368 elements, which correspond to approximately 450 µm elements with respect to the X–Y plane. The composite adherends are composed of 50,000 elements, with each element corresponding to approximately 500 µm in all dimensions (Figure 11).

## 4. Results

**Figure 12:**
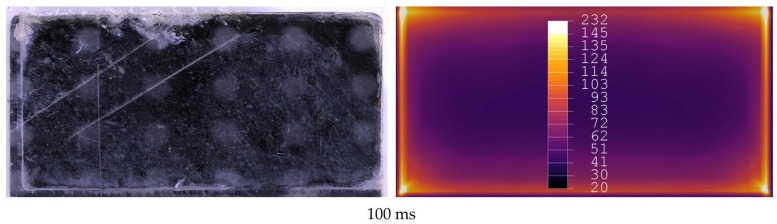
Comparison of fracture surfaces from validation experiments and simulation at 100 ms welding time. The affected area is limited to the edges of the overlap within both images.

**Figure 13:**
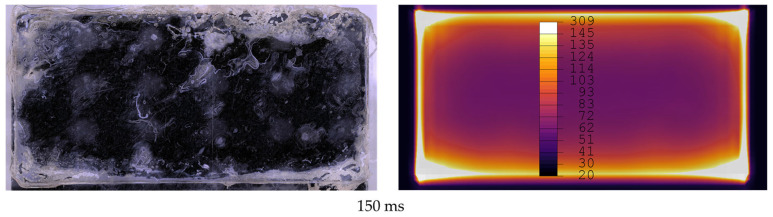
Comparison of fracture surfaces from validation experiments and simulation at 150 ms welding time. The affected area covers all edges and some areas are randomly distributed in the centre of the overlap for validation experiments. Simulation does not show affected areas in the centre.

**Figure 14:**
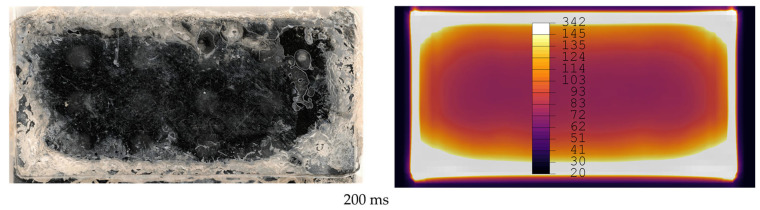
Comparison of fracture surfaces from validation experiments and simulation at 200 ms welding time. The affected area gradually grows from the edges towards the centre with randomly distributed areas closer to the centre of the overlap. Simulation only shows gradual growth of affected areas from edges to centre.

**Figure 15:**
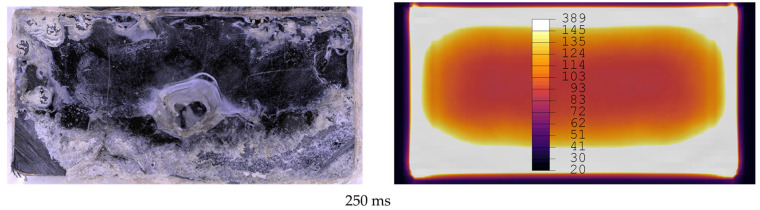
Comparison of fracture surfaces from validation experiments and simulation at 250 ms welding time. The affected area gradually grows from the edges towards the centre with randomly distributed areas closer to the centre of the overlap. Simulation only shows gradual growth of affected areas from edges to centre.

**Figure 16:**
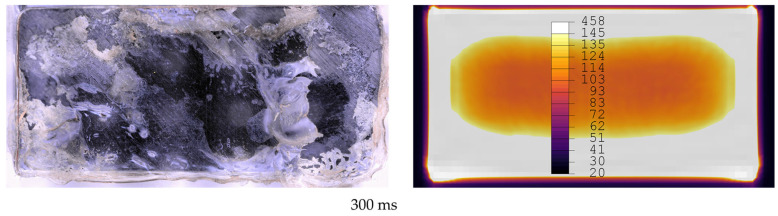
Comparison of fracture surfaces from validation experiments and simulation at 300 ms welding time. Unaffected areas are present only in the centre of the overlap. Simulation shows larger area in the centre as unaffected.

**Figure 17:**
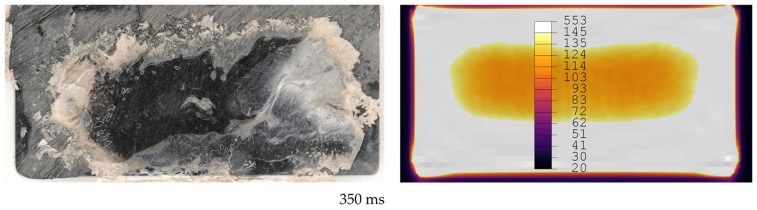
Comparison of fracture surfaces from validation experiments and simulation at 350 ms welding time. Unaffected areas are present only in the centre of the overlap. Simulation shows larger area in the centre as unaffected.

Figure 12, Figure 13, Figure 14, Figure 15, Figure 16 and Figure 17 show fracture surfaces of lap shear coupons from experiments and corresponding temperature plots of the simulation results at the weld interface at the same welding time. In the images showing the simulation results, white areas are linked to a temperature of 145 °C or higher, and the dark centre area indicates the (colder) amorphous, hence transparent energy director.

**Figure 18:**
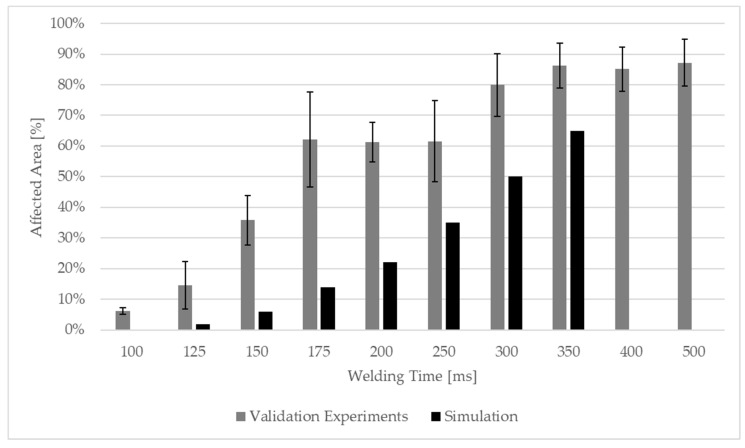
Average affected area of static validation experiments and simulation in percentage. Affected area gradually increases until 300 ms, with plateau from 175 to 250 ms. After 300 ms no further increase in welded area observed. Error bars display standard deviation calculated from six welding experiments per welding time.

Figure 18 shows the amount of affected energy director displayed as a percentage of the total actual overlap area. For the equivalent temperature distributions from the simulation, the area above 145 °C (glass transition temperature [22]) is considered.

**Figure 19:**
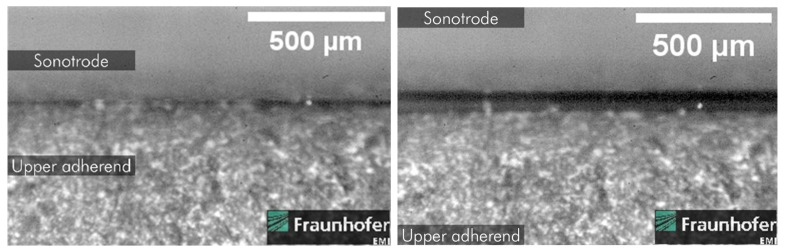
Lifting of the sonotrode from upper adherend observed by high-speed camera recordings at 1 × 10^6^ FPS [18].

**Figure 20:**
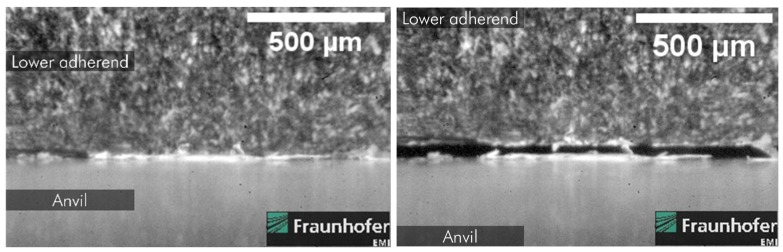
Lifting of the lower adherend from the anvil observed by high-speed camera recordings at 1 × 10^6^ FPS [18].

Figure 19 and Figure 20 show images taken from the high-speed camera recordings at 1 × 10^6^ FPS. They show loss of contact between the sonotrode from the upper adherend and the lower adherend from the anvil, respectively. As already pointed out by us in an earlier publication [18], this loss of contact also occurs between the adherends and the energy director.

**Figure 21:**
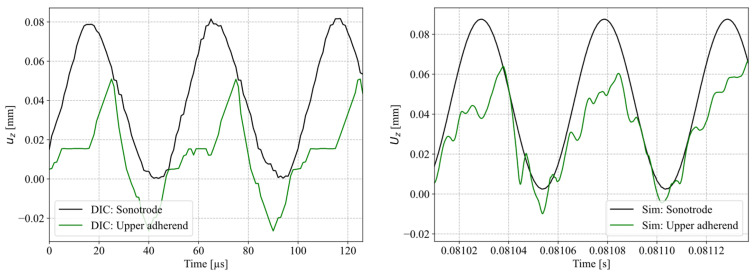
Vertical translation of sonotrode and upper adherend measured at 1,000,000 FPS and estimated via digital image correlation (**left**), and vertical translation of sonotrode and upper adherend from simulation (**right**). Note: due to hardware limitations with respect to recording duration, DIC of data captured at 1,000,000 FPS only allows for relative motion tracking. Therefore, the lowermost sonotrode displacement was defined to be u_z = 0.

Figure 21 compares the vertical translation of the sonotrode and the upper adherend derived from the DIC of the high-speed camera recordings and the simulation. Both graphs show a harmonic oscillation of the sonotrode at the pre-defined amplitude. On the contrary, the translation of the upper adherend is subject to randomness showing random contact of the upper adherend with the sonotrode and the lower adherend. Contact with the lower adherend happens at the lowest displacement of the upper adherend. Due to hardware limitation only the interface between the sonotrode and upper adherend can be tracked within one recording. Negative displacements appear due to the relative nature of the data as it only reflects relative values where the lowest sonotrode position is set to 0 mm.

## 5. Discussion

### 5.1. Validation of the Model

The figures show a good qualitative correlation between the simulation and the actual welding experiments. Although actual temperatures were not been measured during the welding experiments, the change in appearance (e.g., air bubbles or change in colour due to recrystallisation) of the energy director can be used as an indicator that the energy director reached at least glass transition temperature. Actual temperature measurements, e.g., by placing thermocouples in the interphase, were considered, but as thermocouples were likely to influence the welding process by acting as an energy director the comparison to simulation results was less equivalent [23]. A differentiation between areas where glass transition, crystallisation temperature or melting temperature was reached is very difficult as those areas appear almost equally when analysing the fracture surfaces. As expected and observed in previous work [3,23], melting of the energy director starts at the edges of the adherends while in the later stages of the processes the centre of the energy director is also molten. As initial melting of the energy director relies on nucleation hot spots, that are subject to randomness, melting in the centre of the weld area was also present early in the process. Regarding the analysis of fracture surfaces from validation experiments, it has to be noted that even for 500 ms welding time still less than 100% of the overlap area was classified as affected area. The reason for this classification could be the lack of proper consolidation as no consolidation time was applied. Therefore, the adherends are not joined properly but part of the energy director only sticks loosely to the adherend’s surface. During mechanical testing adherends and the energy director are torn apart, leading to the potential movement of parts of the energy director. Because in some areas the energy director only sticks to the surface of the adherends, it leaves no visible marks on the surface and the area seems unaffected. This makes the analysis of fracture surfaces more difficult and can lead to a categorisation of affected areas as unaffected. Therefore, it is likely that for higher welding times the affected overlap areas are larger than assessed.

**Figure 22:**
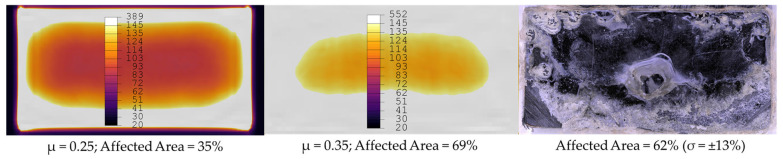
Affected area of simulation using a frictional coefficient of µ = 0.25 (**left**) and µ = 0.35 (**middle**), and the validation trials (**right**) at 250 ms welding time. The frictional coefficient of 0.25 shows underestimation while 0.35 shows close correlation with static welding trials.

As visible in Figure 18 the simulation shows lower affected areas overall compared to the static welding experiments at the same weld time. This difference could be linked to uncertainties in the frictional coefficient implemented in the model. Although the coefficient was determined using an appropriate test specification, the test itself is very sensitive to human factors and the applied normal force (FN) of 21 N corresponding to a pressure of 0.005 MPa is much lower than the welding pressure of 1.6 MPa applied in the welding process. This can lead to the uncertainty of the frictional coefficient. In underestimating the frictional coefficient, the frictional heat dissipation at the beginning of the process is also affected. A sensitivity analysis was performed by implementing different frictional coefficients, with the result that the frictional coefficient indeed influences the resulting temperatures. Figure 22 compares the resulting affected area of a simulation using frictional coefficients of µ=0.25 (left) and µ=0.35 (middle), and the validation trials (right) at 250 ms welding time. The figure shows that a frictional coefficient of 0.25 leads to underestimation of the affected area, whereas a frictional coefficient of 0.35 leads to a result very close to the validation trials.

Another factor which can explain the difference in heating between simulation and static welding experiments is the high standard deviation when determining the neutral position (Figure 7). But as no sensitivity analysis was performed on this parameter the effect cannot be quantified.

Furthermore, it has to be mentioned that the simulation does not incorporate any plasticity and therefore no vertical downward movement of the sonotrode. However, in Figure 6 the data from the high-speed camera (blue) shows a downward movement starting around 200 ms welding time, while the implemented movement of the simulation (black) does not reflect this. This vertical downward movement of the sonotrode is the result of the melting of the energy director and the resulting polymer squeeze flow. It can be argued that this leads to more uniform contact between the adherends and the energy director, resulting in more heat generation as well as reducing the volume of polymer in the welding interface. Both effects can contribute to faster heating and a more extensively affected energy director being observed on the fracture surfaces, compared to the results of the simulation.

Figure 21 also indicates that the movement of the upper adherend in the model shows the same behaviour as observed in high-speed camera recordings. It is important to note that the movement of the adherends and the energy director in the simulation is not a result of a pre-defined input (e.g., as an analytical term) but the implemented mechanical model and the definition of the neutral position of the sonotrode. Furthermore, the resulting heat dissipation and consequent observed temperatures are a result of coupling the mechanical model with the implemented material model.

### 5.2. Neutral Position of Sonotrode

As shown in Figure 6, during the beginning of the welding process the neutral position, which refers to the point of the sonotrode sine wave vibration where the amplitude is zero, increases, staying at a constant level for about 100 ms before decreasing until the end of the welding process. This observation is confirmed by the laser displacement sensor measurements in Figure 7, where a similar behaviour can be observed.

**Figure 23:**
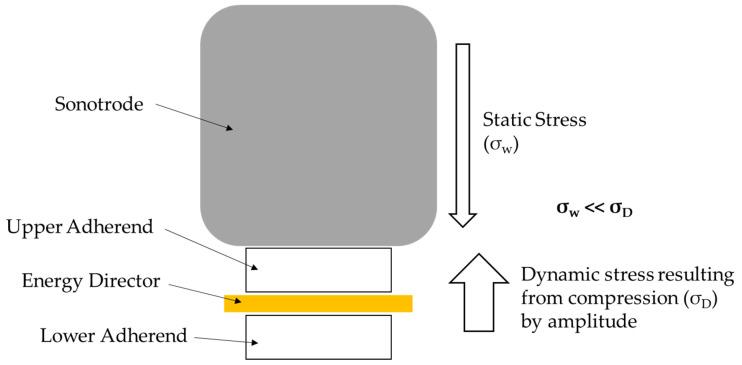
Active stresses in the ultrasonic welding process and their balance.

This higher neutral position compared to the start of the welding process can be explained by the static and dynamic stresses during the welding process [18]. Figure 23 illustrates the different stresses present in the process.

Levy et al. [3] already showed that the resulting dynamic stresses in the material, resulting from the compression of the material to the full extent of the amplitude, are significantly higher than those resulting from the static force.

(12) $$\left|\sigma_{D}\right|=E_{ED}\frac{a}{h_{ED}}+E_{AD}\frac{a}{{2h}_{AD}}.$$

The vertical dynamic stress σ_D_ can be calculated based on Equation (12), where E_ED_ and E_AD_ represents the stiffness of energy director and adherend respectively, the vibration amplitude, h_ED_ as the thickness of the energy director, and h_AD_ as the thickness of the adherends [3].

(13) $$\left|\sigma_{w}\right|=\frac{F_{w}}{A}.$$

Using the mechanical properties and dimensions of the materials and the welding parameters, this provides a dynamic stress of 81 MPa. The stress induced by the static welding force can be calculated based on Equation (13) using F_w_ as the static welding force (500 N) and A as the welding overlap (12.5 × 25 mm^2^), leading to a static stress of 1.6 MPa. Hence, the dynamic stresses are almost two orders of magnitude larger than the static stresses.

The LMPAEK material used as an energy director has a compressive strength of ca. 100–120 MPa according to the material supplier. Comparing the resulting stresses from the previous calculation and the material data for the LMPAEK material it can be noted that the dynamic stress is in the same order of magnitude as the compressive strength of the material. Therefore, we argue that the material is not compressed to the full extent of the amplitude, as this eventually can lead to yielding of the material which usually does not occur in ultrasonic welding. In contrast, the higher neutral position leads to a balancing of static and dynamic stresses as the dynamic stress is reduced because the material is only compressed to a fraction of the nominal amplitude. This higher neutral position was implemented in the simulation based on the data from the laser displacement sensor (Figure 7).

The implementation of the higher neutral position contrasts with previous simulation approaches in the literature, both implicit and explicit. Although some previous studies, e.g., by Zweifel et al. [16] or Tan et al. [14], use actual vibration data instead of the nominal sonotrode vibration as input, the vibrations were still applied to the initial neutral position leading to full compression of the material to the extent of the amplitude. Moreover, vibrations are most often measured not at the tip of the sonotrode but at other points in the welding stack. Therefore, the presented explicit simulation reflects the actual physical behaviour during the welding process and can lead to a more accurate prediction of the heating in the welding interphase.

### 5.3. Hammering Effect

This higher neutral position also implies a new understanding of the hammering effect. The previous understanding of the hammering effect describes the effect as partial loss of contact of the sonotrode and the upper adherend and subsequently only partial transmission of welding energy into the adherends and the energy director. Therefore, it was characterised by a constant hammering coefficient, referred to as α_h_ by Levy et al. [3].

We argue that this loss of contact is not limited to the interaction of the sonotrode and the upper adherends as shown in Figure 20 but also applies to the interaction of the lower adherend and the anvil, shown as well in Figure 20, and the adherends and the energy director. As well as Figure 20, evidence to support this statement is already presented in one of our earlier publications [18].

The previously described higher neutral position of the sonotrode leads to a gap between the sonotrode and the anvil (t_n_) of 43 µm, as measured by the laser displacement sensor (Figure 7). This allows for less constraint vertical movement of the adherends and the ED. This effect is visible in Figure 21 where the upper adherend is not showing a harmonic vibration in sequence with the sonotrode, but rather random movement between the sonotrode and the lower adherend. We observed differences in the curves in Figure 21 mainly attributed to difference between the neutral position implemented in the simulation (45 µm) and the measured neutral position within the single high-speed recordings (67 µm).

In contrast to the previous work of Levy et al. [3] where the hammering effect was considered as a constant factor, our work incorporates the hammering effect as part of the physical representation of the movement of the adherends and the energy director. This adds complexity to the simulation model, requiring more computational resources but increasing the accuracy of predictions about the ultrasonic welding process.

### 5.4. Limitations of the Simulation Model

Nevertheless, the model poses some limitations. In general, it has to be noted that the model is only valid until one of the materials, presumably the energy director, starts to melt as the implemented material models do not cover the molten stage of the materials. For example, the relaxation modulus which was derived from the DMA was only implemented until 275 °C, as performing the DMA in an axial loading condition for higher temperatures proved to be very difficult. Furthermore, the model does not incorporate any plasticity which means polymer squeeze out does not take place. Polymer squeeze out, as it happens in the actual ultrasonic welding process, affects the interphase temperatures, squeezing hot polymer in other areas of the welding overlap or out of the welding interphase.

Furthermore, through-the-thickness heating, especially of the upper adherend as it was observed in previous research, cannot be explained by the model. Most likely the homogeneity approach, where matrix and fibre are not simulated as individual elements but as one material using the rule of mixture, prevents any bulk heating in the upper adherend. To allow frictional heating, relative movement between fibre and matrix is necessary. However, as relative movement is not allowed within the adherends themselves but only between the adherends and energy director, bulk heating in the adherends can only happen once the glass transition temperature is reached. Compared to the adherends, the energy director represents only a small volume. Therefore, it heats up very fast due to frictional heating. In the adherends, heat can be conducted into the different layers or outside the welding area, which generally leads to lower heating rates. Once the glass transition temperature is reached, viscoelastic heating starts to become present. In actual welding processes it is suspected that relative movement between polymer and fibre can appear due to imperfections or defects in the material (e.g., defects induced by mechanical processing or small delaminations) which are always present in actual composite parts, leading to frictional heating and earlier viscoelastic heating within the adherends and as a result, more through-the-thickness-heating occurs. As an example, the technology of vibrothermography takes advantage of this effect to inspect CFRP parts: a CFRP component is excited, e.g., by a piezoelectric transducer, and heating in the part is observed using an infrared camera to locate defects in the component [24].

The current model is characterised by long calculation times of several days for a welding time of 350 ms. At the same time, it uses a significant amount of computation resources. The computation time is mainly driven by the mesh density in certain areas of interest and the lack of mass scaling, such as the area of the energy director to represent the actual welding process as close as possible. Moreover, explicit simulation generally consumes more computation time. To make the simulation model more feasible, simplifications should be implemented to reduce computation times.

## 6. Conclusions

This study investigated how a new explicit modelling approach for the static ultrasonic welding process can more accurately represent the welding process by including a more accurate representation of the hammering effect and frictional phenomena.

We show that an explicit time-domain model is able to represent the static welding process of unidirectional materials using a film energy director. Validation experiments show that the way the welding interface heats up, mainly the energy director, is in close correlation with the simulation results. However, the sensitivity to material input data is high; for instance, the frictional coefficient can significantly influence the results.

The study also shows that due to the high dynamic stresses relative to the stresses from the static welding force, the neutral position of the sonotrode increases during the beginning of the welding process. This allows free, less constraining movement of the adherends and the energy director. This leads to loss of contact not only between the sonotrode and upper adherend (current definition of hammering), but also between the energy director, the adherends and the anvil.

To the authors’ knowledge, the study is the first time that this higher neutral position of the sonotrode was then implemented in an explicit simulation of the static ultrasonic welding process. In combination with the physical representation of the movement of the adherends and the energy director, a more realistic hammering effect was incorporated as a physical cause for heating due to the conservation of momentum and balancing of forces.

Nonetheless, the model also shows some limitations. It is only able to show good correlation with physical experiments upon melting of the materials, presumably the energy director. The implemented material models are not valid beyond melting and the model does not include plasticity to represent polymer and fibre squeeze out. Furthermore, the model is still not able to explain through-the-thickness heating of the adherends due to the applied homogenisation approach to model the composite adherends.

## Figures and Tables

**Table 1:** Thermal conductivity used for modelling of VICTREX LMPAEK™ [20] and Toray Cetex^®^ TC1225, provided by manufacturer.

Material	Temperature	$\lambda_{eq.xx}$	$\lambda_{eq.yy}$	$\lambda_{eq.zz}$
		[mW mm^−1^ K^−1^]		
Toray Cetex^®^ TC 1225	20 °C	4.54	2.67	0.74
	200 °C	6.35	3.69	0.91
VICTREX LMPAEK™	20 °C	0.24		
	250 °C	0.284		

## Data Availability

The original contributions presented in this study are included in the article. Further inquiries can be directed to the corresponding author.
